# On the nose: genetic and evolutionary aspects of smell

**DOI:** 10.1186/s13323-015-0021-3

**Published:** 2015-02-17

**Authors:** Mark A Jobling

**Affiliations:** Department of Genetics, University of Leicester, University Road, Leicester, LE1 7RH UK

Among my Christmas presents this year was some perfume – Acqua di Parma, in a beautiful cylindrical buttercup-yellow box. It was from my son, who since his transition to adulthood has developed an interest in such things, and in return we gave him (as requested) Terre d’Hermès. To my ill-educated nose, both smell pretty good - but there’s a more expert source to turn to for an opinion. This is *Perfumes: the A-Z Guide*, by Luca Turin and Tania Sanchez [1]. Behind its unpromising title lies an entertaining, witty and informative book. The authors write with withering style about the scents they most dislike. So it’s with trepidation you look up the perfume you’ve acquired – luckily those mentioned above both rate a respectable three out of five stars. One-star reviews include ‘smells like a New York sidewalk in July’, ‘less a fragrance than a headache force-field’, and ‘useful as a contraceptive, but little else’.

The ~1800 perfumes sniffed for *The A-Z Guide* are testament to the lengths to which we humans will go to make us smell like something else. We wash ourselves with soaps and shampoos, anoint our sweatiest bits with deodorants and antiperspirants, and then spray on expensive cocktails of scented chemicals and natural extracts. This is curious, because it appears that nature’s clear intention was for us to smell abundantly of ourselves.

When our ancestors lost their body hair, they retained the associated sebaceous glands designed to anoint each hair with water-repellent secretions. In fact, we have denser aggregates of such glands than almost any other mammal. In addition, we each have 3 million sweat glands capable of exuding 12 litres of cooling fluid daily. There are two varieties – eccrine, which secrete 99% water, and apocrine, which secrete an oily fluid including proteins, lipids, fatty acids and steroids. The apocrine glands are confined to the hairy parts of the body, including the genital area and armpits (axillae). Indeed, in most humans the axillary density is so great that the array of glands is considered an organ. So, with our inherently smelly sebaceous secretions, and our apocrine sweat, made odorous by skin bacteria, we are without doubt the ‘scented ape’ [2].

Scented humans may be, but some are more scented than others. A few unfortunate people are homozygous for mutations in the gene encoding an enzyme, flavin-containing monooxygenase 3 [3], whose job is to metabolise amino-trimethylamine, produced by bacterial action in the gut. In the absence of the enzyme, the chemical is secreted in the sweat, urine and breath – its smell, reminiscent of decaying fish, makes the lives of sufferers very difficult indeed. In the general population travellers and anthropologists have remarked upon differences between the groups they encountered. Some of the early anecdotal accounts are, to modern minds, highly derogatory, and do not bear repeating. The major observation, however, seems real and evolutionarily interesting: in general, East Asians are less smelly than everyone else. This is connected to the number of apocrine glands in the axillary organ; while Europeans and Africans have glands packed so closely that they resemble a sponge, in Koreans (for example) they are either spread thinly or absent altogether [2].

The genetic basis of apocrine gland density is unknown, but genetics has illuminated population differences in odour via the seemingly unrelated subject of earwax. There are two kinds – the grey and flaky ‘dry’, prevalent in East Asians, and the yellow and waxy ‘wet’. A single nucleotide variant in the genome is responsible for this difference [4] – individuals who carry an A nucleotide at the relevant position in both copies of their *ABCC11* gene (AA homozygotes) have dry earwax, while GA heterozygotes or GG homozygotes have the wet variety. The population distribution suggests positive selection for the A-allele in Asia, but earwax itself seems an improbable candidate. However, earwax emerges from specialised apocrine glands, and analysis of sweat from people carrying different genotypes indicates that the *ABCC11* A variant is also responsible for reduced axillary odour [5], thanks to a failure to transport a smelly-molecule precursor into the sweat [6]. How selection came to act so strongly on this variant is not clear – perhaps choice of partner (sexual selection) was influenced strongly by odour, though why this should be so in some parts of the world but not in others, is puzzling.

There are two sides to smell, of course – as well as production, there is perception, and here most emphasis has been on differences between individuals. At one extreme, some people are born with no sense of smell at all (anosmia). Kallmann Syndrome is usually caused by mutations in the *KAL1* gene on the X chromosome, and is often associated with complete anosmia in the male sufferers [7]. This phenotype arises from the failure of neurons in early development to migrate to form the olfactory bulb, where the sense of smell arises; the neurons also fail to reach their next destination, the hypothalamus, with the result that gonadotropin-releasing hormone is not produced. This in turn leads to failure of puberty, and to infertility.

Odour detection is mediated through olfactory receptors (ORs) in the cell membranes of olfactory neurons, encoded by a family of over 300 genes [8]. A combinatorial code of different ORs interacts with odorant molecules, so mutations involving OR genes could lead to specific anosmias – the inability to smell particular odorants. Indeed, genome-wide association studies have found variants within OR gene clusters linked to sensitivity to methanethiol (secreted in the urine after eating asparagus) [9], androstenone (produced in human sweat, by truffles, and by pigs in the mating season) [10], and also to the floral-smelling compound β-ionone [11]. The latter probably explains why some people cannot smell β-ionone-rich freesias [12].

So, what’s the purpose of human scent and our sense of smell? Other animals use scented chemicals (pheromones) to attract the opposite sex, and to indicate fertility – they induce stereotypical behaviours, as anyone who has owned a cat or dog in heat will know. Humans become highly scented animals when they reach sexual maturity, and poets from Catullus to Herrick have written with passion about the fragrances of their lovers. In the nineteenth century rustic Austrian girls used to keep a slice of apple in their armpits during a dance, and would afterwards offer it to their favoured partner to eat, as a token of interest [2]. Despite these sexual connections, compared to other animals, the human sense of smell is of little biological use. The relative proportion of the brain occupied by smell has decreased steadily in the primate lineage from lemurs to humans, and we lack the vomeronasal organ – the ‘second nose’ above the palate that causes our cats and dogs to act in response to pheromones. Plenty of websites offer fragrances such as ‘Alpha Dream’ and ‘PheroMen’, with promises of instant sexual irresistibility. But, despite reports of odour-mediated menstrual synchrony in female roommates [13], there is little evidence that human pheromones exist.

It seems that our production and perception of smell may be evolutionary vestiges, relegated when we stood upright and our visual systems became of primary importance, and when the need for pair-bonding made advertising female receptiveness disadvantageous. Yet scent enriches our lives, and its animal roots are never far away – among the ingredients of fine perfumes are substances scraped from the anal glands of indignant civet cats, or extracted from the musk glands of rutting male Himalayan deer.
